# A Review of Various Attempts on Multi-Functional Encapsulation Technologies for the Reliability of OLEDs

**DOI:** 10.3390/mi13091478

**Published:** 2022-09-06

**Authors:** Yongmin Jeon, Hyeongjun Lee, Hyeunwoo Kim, Jeong-Hyun Kwon

**Affiliations:** 1 Department of Biomedical Engineering, Gachon University, 1342 Seongnam-daero, Sujeong-gu, Seongnam-si 13120, Korea; 2 Department of Display and Semiconductor Engineering, Sun Moon University, Asan 31460, Korea

**Keywords:** thin-film encapsulation, organic light-emitting diode, environmental reliability, atomic layer deposition, transparent flexible display

## Abstract

As the demand for flexible organic light-emitting diodes (OLEDs) grows beyond that for rigid OLEDs, various elements of OLEDs, such as thin-film transistors, electrodes, thin-film encapsulations (TFEs), and touch screen panels, have been developed to overcome OLEDs’ physical and chemical limitations through material and structural design. In particular, TFEs, which protect OLEDs from the external environment, including reactive gases, heat, sunlight, dust, and particles, have technical difficulties to be solved. This review covers various encapsulation technologies that have been developed with the advent of atomic layer deposition (ALD) technology for highly reliable OLEDs, in which solutions to existing technical difficulties in flexible encapsulations are proposed. However, as the conventional encapsulation technologies did not show technological differentiation because researchers have focused only on improving their barrier performance by increasing their thickness and the number of pairs, OLEDs are inevitably vulnerable to environmental degradation induced by ultraviolet (UV) light, heat, and barrier film corrosion. Therefore, research on multi-functional encapsulation technology customized for display applications has been conducted. Many research groups have created functional TFEs by applying nanolaminates, optical Bragg mirrors, and interfacial engineering between layers. As transparent, wearable, and stretchable OLEDs will be actively commercialized beyond flexible OLEDs in the future, customized encapsulation considering the characteristics of the display will be a key technology that guarantees the reliability of the display and accelerates the realization of advanced displays.

## 1. Introduction

Encapsulation technology has been extensively applied and developed to protect products from the external environment; from food to electronic devices [1,2,3,4]. In the early days, the understanding of permeation barrier technology was based on food and medical packaging. As organic semiconducting devices began to be developed, a much higher level of gas barrier coating technology was required to secure reliability. From physical vapor deposition (PVD) to chemical vapor deposition (CVD) to atomic layer deposition (ALD), breakthroughs in deposition technology have led to significant improvements in gas barrier technology.

**Figure 1:**
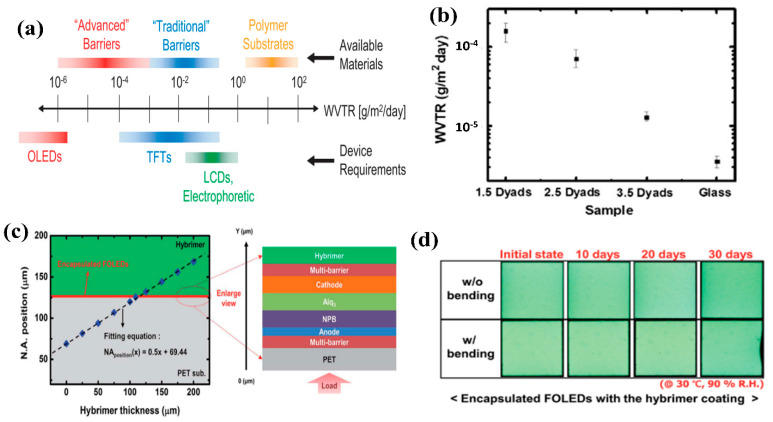
(**a**) WVTR requirements for the realization of flexible electronic devices and barrier performances of the available materials. Reproduced with the permission of Reference [4]. Copyright 2013, Elsevier. (**b**) Average WVTR values according to the number of dyads of Al_2_O_3_/organic multi-barrier and glass. Reproduced with the permission of Reference [19]. Copyright 2006, Elsevier. (**c**) Neutral axis (NA) position in the flexible OLED structure with increasing buffer thickness. (**d**) Emitting cell images of NA-controlled OLEDs before and after bending test. Reproduced with the permission of Reference [24]. Copyright 2016, Royal Society of Chemistry.

Various organic electronic devices (OEDs), such as organic light-emitting diodes (OLEDs), organic solar cells (OSCs), and organic thin-film transistors, have been widely studied due to their various merits [5,6,7,8,9,10,11,12]. Although the OEDs composed of organic thin layers and metal thin films were ultrathin and flexible, they were easily degraded by exposure to external environments due to the strong chemical reaction of water vapor and oxygen, resulting in degradation in their lifetimes and efficiencies. In particular, the widely quoted requirement for water vapor transmission rate (WVTR) for an OLED lifetime of >10,000 h is known to be less than 10^−6^ g·m^−2^·day^−1^ (Figure 1a) [4,13]. Therefore, encapsulation technologies that fulfill WVTR requirements are required to guarantee the long-term reliability of OEDs and have been actively studied, from rigid glass-lid encapsulation to flexible thin-film encapsulation (TFE), depending on device type. In flat-panel displays, the conventional encapsulations using glass or metal lids, which were attached to devices using ultraviolet (UV) sealant, were mostly used to ensure the reliability of organic electronic devices. However, as the demand for flexible displays grows, so does the demand for next-generation displays. Conventional encapsulation technologies, such as glass-lid and single-layer inorganic encapsulation, have become more difficult to use as flexible encapsulations due to their rigidity. In addition, as flexible displays use a flexible and thin plastic film instead of a thick and fragile glass substrate, moisture and oxygen permeating through the substrate must be considered. In general, it is known that the polymer substrate has a WVTR in the range of 1 to 100 g·m^−2^·day^−1^ (Figure 1a). Therefore, in order to realize a flexible OLED, it is necessary to consider not only moisture and oxygen entering the top and side of the OLED, but also moisture and oxygen entering the substrate, so it is necessary to apply an encapsulation technology that can achieve a WVTR of 10^−6^ g·m^−2^·day^−1^. Consequently, flexible encapsulations based on thin films, called TFE or Barix^TM^ encapsulations, were developed through the combination of inorganic and organic layers [14,15,16,17,18,19,20,21,22]. The general multi-barrier system was formed by alternately stacking a brittle inorganic film, which acts as a major barrier to moisture and oxygen, and a flexible organic film, which reduces internal stress, planarizes the surface of the inorganic film, and complicates the gas diffusion path. The initial TFEs were complex, with more than three dyads (here, one dyad means an inorganic/organic multi-layer), and exhibited poor barrier and mechanical properties [19,20,23,24]. However, as previously mentioned, as high-tech deposition techniques based on CVD and ALD were developed, inorganic gas diffusion barriers (GDBs) were found to have excellent barrier properties at a thickness of less than 100 nm [19,25,26,27,28,29,30]. As the barrier properties of inorganic GDBs were gradually improved, the TFE structure became simpler and its total thickness was reduced. In addition to the improvement of barrier performances, the mechanical flexibility of TFEs has been improved through the use of inorganic GDBs, which are called nanolaminates [31,32,33,34,35,36,37,38,39,40,41,42,43,44,45,46]. Beyond a simple inorganic–organic multi-barrier structure as a GDB, the functional design of TFEs combining functional layers enables OEDs to be environmentally and mechanically reliable, thereby relieving the residual stress as well as effectively blocking reactive gases and harmful UV light [12,18,25,32,45,46,47].

This review paper highlights the recent progress in TFE technologies suitable for the realization of highly reliable flexible OLEDs fabricated on flexible substrates, covering the challenges of developing alternative transparent flexible encapsulation technologies that possess the functions and importance of TFEs in terms of device reliability and robustness. Recently, substantial efforts have been made to develop functional TFEs as well as to diminish the total thickness and the number of dyads of TFEs without degrading the barrier property, to achieve the commercialization and reliability of flexible displays. Beyond flexible OLEDs, corrosion-resistant and mechanically resistant encapsulations need to be applied for the washing of wearable displays, in which deformable OLEDs are implemented on a fabric or fiber substrate.

## 2. Highly Impermeable and Flexible TFE Using Stress-Robust Techniques

The inorganic layer’s role as a practical gas barrier against water and oxygen permeation has triggered numerous studies to develop an excellent gas-impermeable inorganic layer. Although various inorganic layers have been developed based on various deposition methods, including thermal evaporation, sputtering, and CVD layers, with the advent of ALD deposition, inorganic GDBs have evolved one step further. Among inorganic layers, studies on Al_2_O_3_ films have been actively conducted using sputtering or ALD deposition, as they are simple deposition methods. However, sputtered Al_2_O_3_ films exhibit low film quality with many defects and pinholes due to sputtering being based on PVD, and they are difficult to directly apply to OLED devices due to their exposed plasma and poor barrier performance. In contrast, ALD enables Al_2_O_3_ film to be stably deposited under a low-temperature vacuum chamber and minimizes its defects. Therefore, in recent years, studies on ALD-based GDBs have been actively conducted to create highly impermeable GDBs. ALD enables a low-temperature process that does not cause thermal damage to OLEDs and yields an Al_2_O_3_ thin film that can be easily deposited with ALD, enabling it to be used in various electronic devices as a GDB or gate insulator. However, the single Al_2_O_3_ ALD thin film cannot be used as a GDB for flexible OLEDs that require high barrier performance due to its poor GDB properties and mechanical stability. Therefore, more than three dyads of multi-barriers using Al_2_O_3_ and an organic layer have been fabricated to achieve the WVTR of less than 10^−5^ g·m^−2^·day^−1^ as shown in Figure 1b. Although TFEs achieved better WVTRs by increasing their number of dyads, complex TFE technologies and limited flexibility make this solution difficult to use in TFEs for commercialized flexible OLEDs. To improve the mechanical flexibility of Al_2_O_3_-based TFEs, Han et al. proposed controlling the neutral axis (NA) position by additionally coating a buffer layer into thin-film encapsulated OLEDs (Figure 1c,d) [24]. In pure bending of flexible substrates fabricated on thin films and devices, there is an axis within the plane where the stress and strain are zero. The axis is referred to as the NA. The inserted buffer layer placed the NA in the OLED device, and as a result, the flexibility of the encapsulated OLED was improved. However, the encapsulation structure consists of many layers including a buffer layer, and it required optimization through simulations according to the panel thickness including OLED. Therefore, the development of a new type of inorganic thin film through material and structural design is required to improve the impermeable and mechanical properties while simplifying the TFE structure for use as a flexible TFE.

**Figure 2:**
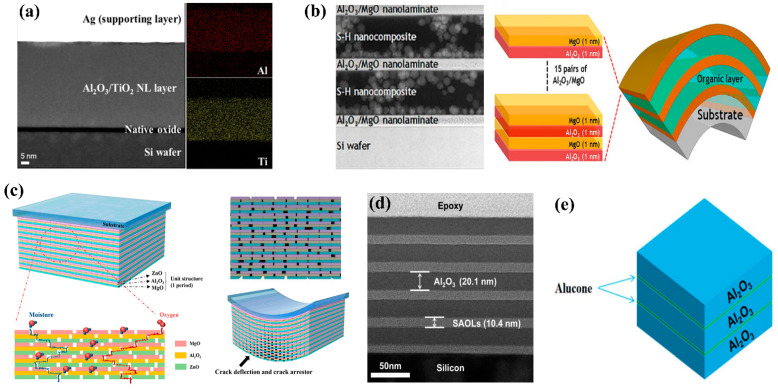
ALD nanolaminate system. (**a**) TEM-EDS mapping images showing amorphous structure of Al_2_O_3_/TiO_2_ NL film without any interfaces. Reproduced with the permission of Reference [33]. Copyright 2014, American Chemical Society. (**b**) Cross-section image of the 2.5 dyads of Al_2_O_3_/MgO nanolaminate/organic layer multi-barrier when the cycle ratio of Al_2_O_3_:MgO is 11:11. Reproduced with the permission of Reference [47]. Copyright 2020, Springer Nature. (**c**) Schematic of the ZnO/Al_2_O_3_/MgO (ZAM) nanolaminate structure with increased lag time (**left**) and bent ZAM film with crack deflection and crack arresting (**right**) using a defect-decoupling system. Reproduced with the permission of Reference [41]. Copyright 2017, American Chemical Society. (**d**) Cross-sectional transmission electron microscopy (TEM) image of SAOLs/Al_2_O_3_ nanolaminate structure deposited using MLD and ALD. Reproduced with the permission of Reference [45]. Copyright 2017, American Chemical Society. (**e**) Schematic illustration of Al_2_O_3_/alucone 15–2.5 encapsulation layers. Reproduced with the permission of Reference [39]. Copyright 2019, Royal Society of Chemistry.

In recent years, for the fabrication of a highly impermeable and flexible inorganic layer, a nanolaminate technology that alternately stacks different thin films of several nanometers in multiple layers has been proposed, thus suppressing the growth of nano-sized and micro-sized pinholes and voids as well as suppressing the crystalline growth of the fabricated thin film. ALD has enabled the fabrication of ALD nanolaminate structures with thickness in angstrom units and having excellent uniformity within a certain area. ALD nanolaminate structures including Al_2_O_3_ sublayers have shown significantly improved barrier performance compared to single-layer structures, as shown in Table 1. Dameron et al. first proposed the concept of nanolaminates using ALD methods and experimentally proved the superiorities of the multi-layer structures [40]. Since then, various ALD nanolaminate structures have been developed by combining other oxides with the Al_2_O_3_ sublayer. However, although previous nanolaminate structures were fabricated by combining two kinds of sublayers considering the material design, no study has been conducted to optimize the thickness of the nanolaminate and its comprising sublayers. Inorganic GDBs formed on a plastic substrate have WVTR-saturated thickness ranges due to their residual stress build-up [1,41]. Therefore, thickness optimization is required for the fabrication of an outstanding barrier film. In addition, the thickness of the ultrathin sublayers constituting nanolaminate structures is the main factor in optimizing key properties of nanolaminates due to their multi-interfacial structure. Kwon et al. proposed ALD nanolaminate structures based on ZnO, Al_2_O_3_, and MgO sublayers through structural and material design to fabricate mechanically and environmentally robust TFEs (Figure 2c) [41]. ZnO, Al_2_O_3_, and MgO (ZAM) ALD sublayers were used owing to their functions of stress relief, gas permeation barrier, and water absorption, respectively. In addition to the function of each layer, the Al_2_O_3_ sublayer also formed a chemical bond called the aluminate phase at the interface between the ZnO and MgO sublayers, enhancing the barrier and environmental robustness of the nanolaminate structure. Therefore, the ZAM nanolaminate was designed with excellent gas barrier properties, resulting in the WVTR of 10^−5^ g·m^−2^·day^−1^ with 1 nm thick sublayers and a total thickness of approximately 50 nm. Furthermore, the multi-barrier of 1.5 dyads with the structure of a ZAM/organic layer/ZAM resulted in an extremely low WVTR of 2.04 × 10^−6^ g·m^−2^·day^−1^ and mechanical stability that endured a tensile strain of approximately 1%. Furthermore, the ALD nanolaminate structure helped improve the residual stress and mechanical properties of the fabricated thin film in comparison to the Al_2_O_3_ single layer [41,42,46]. Therefore, the optimized nanolaminate structure showed remarkably improved barrier performance and mechanical properties through structural as well as material design in comparison to the Al_2_O_3_ single layer and enabled the simplification of the TFE structure. Most recently, the organic–inorganic nanolaminate barrier was proposed using ALD for inorganic layers and molecular layer deposition (MLD) for inorganic–organic hybrid layers [39,45,48,49,50]. MLD is a technique for forming a hybrid organic–inorganic thin film that shows growth at the molecular level based on successive self-limiting surface reactions, like in ALD. Although the organic–inorganic hybrid film deposited by MLD has a low density, it is soft and flexible, so an organic film can be utilized for encapsulation. The combination of ALD and MLD enables the formation of organic/inorganic multi-layer structures without solution processes such as spin coating or inkjet printing for organic layer formation. In other words, a one-step process in the ALD chamber is possible while maintaining a vacuum atmosphere and drastically reducing the total thickness of the TFE. Kwan et al. reported that the 300 nm thick hybrid nano-laminated multi-layer composed of Al_2_O_3_ and self-assembled organic layers demonstrated a WVTR of 2.99 × 10^−7^ g·m^−2^·day^−1^ at RT before bending and a WVTR of 1.31 × 10^−6^ g·m^−2^·day^−1^ after bending with a bending radius of 10 mm (Figure 2d). Chen et al. demonstrated the barrier properties and mechanical flexibility of TFE based on the alucone film produced using trimethylaluminum and ethylene glycol as precursors (Figure 2e). From nanolaminate technology to the MLD introduced above, many attempts have been made to satisfy the demand for superior encapsulation technology. For the continuous advancement of foldable and rollable displays for commercialization, applying all aspects of stress engineering, including NA control, nanolaminates, residual stress control, and the MLD process, is important.

From nanolaminate technology to the MLD introduced above, many attempts have been made to satisfy the demand for a superior encapsulation technology. For the continuous advancement of foldable and rollable displays for commercialization, it will become more important to apply all aspects of stress engineering, including NA control, nanolaminates, residual stress control, and the MLD process.

## 3. Various Functional TFE Technologies for the Reliability of OLEDs

### 3.1. Heat-Transferable TFE Inserted with Thermally Conductive Films

**Figure 3:**
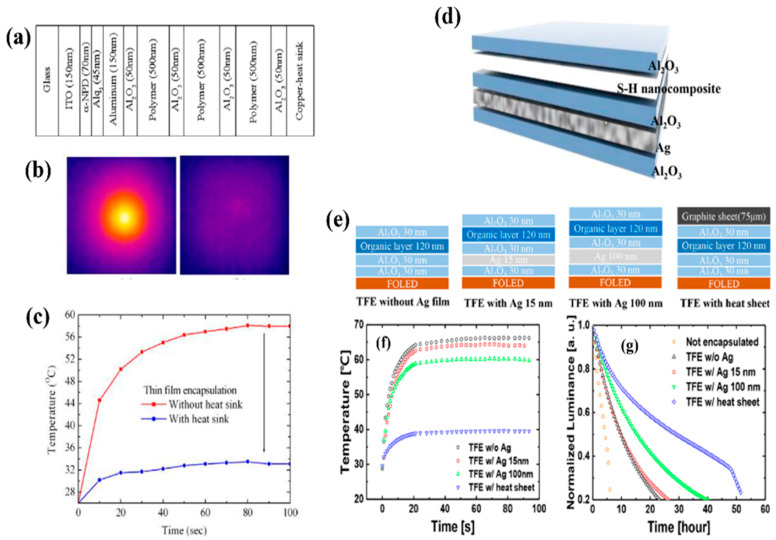
Thermally conductive TFE. (**a**) Schematic of an OLED encapsulated with a TFE heat sheet. (**b**) Infrared (IR) images of encapsulated OLEDs according to the presence of the heat sheet. (**c**) Temperature profiles of the measured maximum surface temperature. Reproduced with the permission of Reference [51]. Copyright 2011, Elsevier. (**d**) Schematic of dielectric/metal/dielectric (DMD)-TFE inserted with a Ag thin film. (**e**) Schematics of various kinds of TFE structures according to the presence and thickness of the Ag thin film and heat sheet. (**f**) Surface temperature profiles of operating flexible OLEDs encapsulated with various TFE structures over time. (**g**) Results of an acceleration lifetime test of flexible OLEDs encapsulated with various kinds of TFE. Reproduced with the permission of Reference [18]. Copyright 2017, American Chemical Society.

In the realization of flexible displays, heat dissipation can be an important issue. As most polymer substrates have thermal conductivity of 0.5 W·mK^−1^ or less, when the luminous efficiency of an OLED device is low or when high luminance needs to be maintained for long periods, the stagnant heat inside the device raises the device temperature and may lead to performance degradation [18,23,51,52,53,54,55]. In particular, when the surface temperature of an OLED is 42 °C or higher, low-temperature burns may occurs when used as a wearable display [56]. The heat generated by electrical device operation causes performance degradation, which seriously affects OLED efficiency and lifetime, serving as a hurdle to the realization of flexible displays. In particular, the thermally insulating properties of TFE hinder heat dissipation in OLEDs. Therefore, in an attempt to dissipate heat inside OLEDs, an attempt was made to improve the thermal degradation by applying a thick heat sink on the encapsulated OLED [51,52]. Park et al. investigated the surface temperature distribution of OLEDs according to the presence of the heat sheet using numerical simulations and experiments (Figure 3a–c) [51]. The copper sheet used for heat dissipation was attached to the TFE layer using a thermal radiation layer. Because TFEs are composed of inorganic/organic layers with low thermal conductivity, a heat dissipation effect by TFE was not expected. However, the attachment of the heat sheet to TFE facilitates heat dissipation of OLEDs. As a result, there was almost no difference in heat transfer performance based on the number of TFE dyads based on the simulation. Although the metal heat sink was effective at dissipating heat in OLEDs, additional use of a thick and opaque heat sink was limited for the realization of transparent flexible OLEDs. Attempts to fabricate a thermally conductive TFE based on thin films were first made by Kwon et al. [23,53]. They tried to develop thermally conductive TFE using a dielectric/metal/dielectric (DMD) structure with a 15 nm thick Ag thin film inserted between Al_2_O_3_ films due to the high thermal conductivity and good flexibility of the Ag thin film (Figure 3d). The DMD structure was mainly used to fabricate a transparent flexible multi-layer electrode. The thickness-optimized DMD-TFE demonstrated an optical transmittance of more than 60%, a WVTR of 8.70 × 10^−6^ g·m^−2^·day^−1^, and good mechanical reliability at a bending strain of 0.41%. However, the heat dissipation effect of the DMD-TFE with 15 nm thick Ag film in OLEDs was very weak due to the scattering of free electrons on the surfaces and grain boundaries. In contrast, DMD-TFE with 100 nm thick Ag film demonstrated a better heat dissipation effect on OLEDs (Figure 3f,g). In addition to the metal insertion, graphene, which possesses higher thermal conductivity than Ag, can also fabricate more thermally conductive TFE. As graphene, which has been attracting attention for a long time due to its excellent thermal and electrical conductivity as well as mechanical durability, comes in the form of a two-dimensional honeycomb lattice nanostructure made of carbon atoms, graphene-based TFEs have been created due to their gas barrier properties and flexibility [6,57,58,59,60]. Graphene is difficult to use alone or as a single layer due to its many defects, but when combined with other films or composing a multi-layer graphene sheet, it facilitates a significant improvement in barrier performance. However, the reported graphene-based TFE did not achieve a WVTR of less than 10^−5^ g·m^−2^·day^−1^ at a level applicable to OLEDs, and there have been no cases in which it was reported as a functional encapsulation film with heat dissipation function, which is thought to be more meaningful.

Although the DMD-TFE encapsulated OLED did not show a significant improvement in its lifetime, the studies on heat-dissipating TFE introduced above will help improve the lifetime and efficiency of OLEDs through follow-up studies on the encapsulation technology combined with new heat-conducting materials by showing their potential and methodologies.

### 3.2. Transparent Conductive Gas Diffusion Barrier

**Figure 4:**
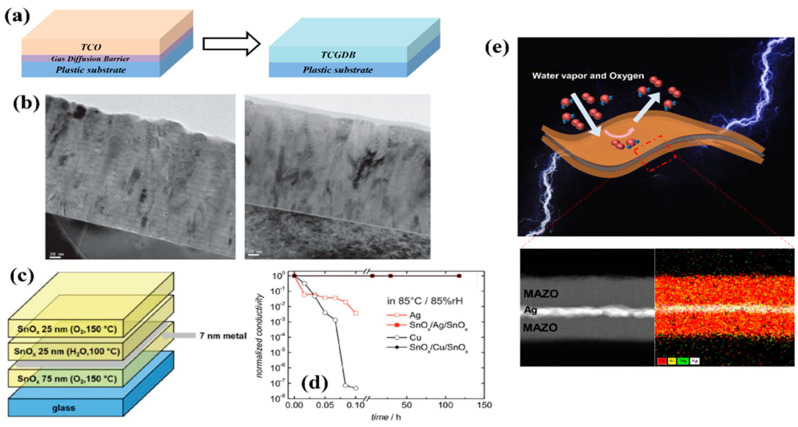
Transparent conductive gas diffusion barrier (TCGDB). (**a**) Schematic of a dual-functioning TCGDB combined with transparent conductive oxides (TCOs) and a GDB. Reproduced with the permission of Reference [25]. Copyright 2018, American Chemical Society. (**b**) Cross-sectional TEM images of a conventional (**left**) and a mixed (**right**) Hf-doped ZnO (HZO) film. Reproduced with the permission of Reference [65]. Copyright 2013, Wiley-VCH GmbH. (**c**) Schematic of the transparent conductive SnOx/Ag/SnOx multi-layer. (**d**) Normalized conductivity of bare metal thin film and SnOx/metal/SnOx multi-layer stored at 85 °C/85% relative humidity. Reproduced with the permission of Reference [30]. Copyright 2017, Wiley-VCH GmbH. (**e**) Schematic of conductive and moisture-resistant Mg- and Al-doped ZnO (MAZO)-based multi-layer electrode (inset: cross-sectional TEM/energy dispersive spectrometry mapping images of the MAZO/Ag/MAZO multi-layer). Reproduced with the permission of Reference [25]. Copyright 2018, American Chemical Society.

Transparent conductive oxides (TCOs) have been developed for the fabrication of transparent optoelectronic devices. Among TCOs developed thus far, sputtered indium tin oxide (ITO) has been widely used as an electrode due to its high optical transparency, electrical conductivity, and excellent chemical stability [25,61,62,63,64]. As such, ITO electrodes have been used as an irreplaceable TCO for a long time. However, there remain aspects of the TCO technologies that require further improvement to achieve technical advances in flat-panel displays and flexible-panel displays. However, although ITO electrodes are good electrical conductors for rigid devices, they show poor GDB properties and flexibility due to their deposition method and crystallinity [25,30,64,65]. If electrodes were moisture-resistant and flexible, indispensable TFE processes for OLEDs could be omitted or simplified. Therefore, OLED devices can achieve higher manufacturing capability and lower manufacturing costs by replacing TFE with an electrode. Recently, to meet the demands for a dual-functioning TCO that serves as both an electrode and a GDB, Chou et al. proposed transparent conductive GDBs (TCGDBs) (Figure 4a) [65]. They developed a conductive Hf-doped ZnO (HZO) film with high conductivity, transparency, and gas barrier performance using ALD at a temperature of 150 °C (Figure 4b). The designed HZO film exhibited good electrical properties as well as a WVTR of 6.30 × 10^−6^ g·m^−2^·day^−1^ with ZnO crystallinity. Since this first report on TCGDBs, Behrendt et al. reported robust transparent and conductive tin oxide (SnO_x_) TCGDBs deposited by ALD at 150 °C to overcome the chemical instability and poor electrical conductivity of ZnO ALD films [30,66]. The developed SnO_x_ achieved a WVTR of 3.10 × 10^−6^ g·m^−2^·day^−1^ at 200 nm and remained stable despite changes in the electrical conductivity within one order of magnitude after a damp heat test for 50 days. In addition, Behrendt et al. fabricated a DMD structure of SnO_x_/Ag/SnO_x_ (SAS) using the developed SnO_x_ to fabricate flexible electrodes (Figure 4c). SAS showed not only excellent environmental stability and sheet resistance of less than 10 Ω/sq., but also feasibility as a transparent electrode in OSCs and OLEDs (Figure 4d). Furthermore, attempts have been made to fabricate functional ALD ZnO films with dual dopant layers at a low temperature of 100 °C [25]. OLEDs are composed of organic layers having low glass transition temperature (T_g_); therefore, the performance of OLED devices degrades at a process temperature of 100 °C or more. In addition, most polymer substrates used for fabricating flexible displays have a low Tg of 100–150 °C, except for PI, so the thin film process must be performed at a temperature lower than T_g_ to ensure no changes in the physical properties of the substrate. The previously reported TCGDBs were deposited at critical temperatures of 150 °C, which can cause the recrystallization of plastic substrates and organic layers. Kwon et al. developed a low-temperature Mg- and Al-doped ZnO (MAZO) layer based on ALD to improve the WVTR, electrical conductivity, and mechanical flexibility of the crystalline ZnO ALD film [25]. By periodically applying Al_2_O_3_ and MgO growth cycles in the middle of growing the ZnO thin film, the crystallinity of ZnO was suppressed, thereby improving the barrier and mechanical properties. Additionally, the electrical conductivity and optical properties were improved due to Mg and Al doping. By fabricating a sandwiched MAZO/Ag/MAZO multi-layer structure that combines this improved MAZO with a thin Ag layer, it achieves a sheet resistance of 5.60 Ω/sq, average transmittance of 89.72% in the visible range, and a WVTR in the order of 10^−5^ g·m^−2^·day^−1^ (Figure 4e). In addition, the MAM electrode used as the anode in OLEDs helped to improve the lifetime of OLEDs in comparison to the ITO electrode with identical thickness. As demonstrated by the previously reported results, further improvement of reliable and flexible TCGDBs will drive the realization of low-cost and high-yield flexible displays.

### 3.3. Functional Encapsulation with UV and Heat Rejection Capabilities

**Figure 5:**
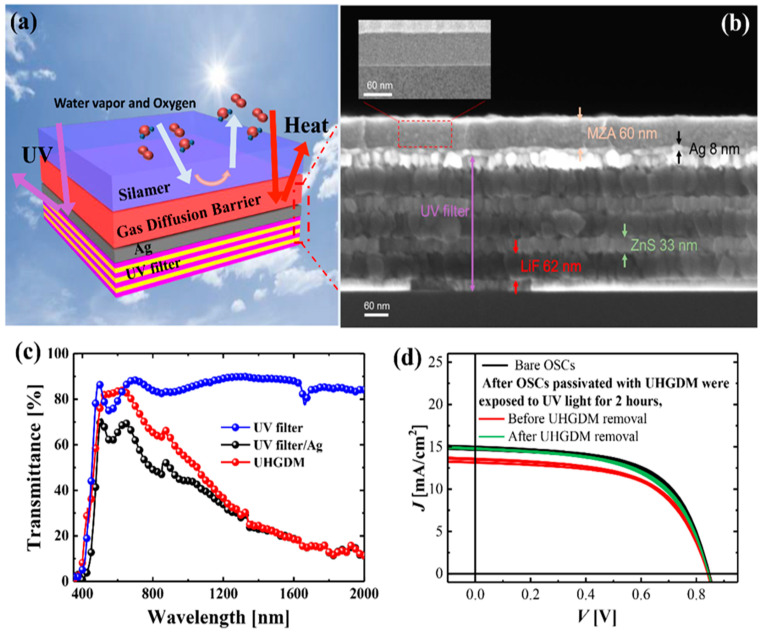
Gas diffusion multi-barrier with UV and heat rejection capabilities. (**a**) Schematic of the UV- and heat-reflective gas diffusion multi-barrier (UHGDM). (**b**) Cross-sectional SEM image of the UV filter/Ag/GDB structure. (**c**) Spectral transmittances of each multi-layer structure in the 350–2000 nm wavelength range. (**d**) Current–voltage characteristic curves of OSCs according to UHGDM removal after UV light exposure for 2 h. (The evaluated OSCs were encapsulated with a glass lid). Reproduced with the permission of Reference [7]. Copyright 2019, American Chemical Society.

TFEs were proposed with an inorganic/organic multi-layer structure due to their slight flexibility and thinness to replace rigid and thick glass encapsulation. The main function of the conventional encapsulation methods is to prevent reactive gases (e.g., oxygen and water vapor) that are harmful to OLEDs from damaging flexible organic electronics. Therefore, TFEs have to be resistant to mechanical stress to avoid mechanical cracking induced by bending or folding so they can guarantee the reliability of OLEDs. However, thin-layered encapsulations can be fabricated with various ultrathin layers. Therefore, TFEs can provide various functions according to the use purpose. Organic electronics that are continuously exposed to the outdoors, such as mobile phones, automotive head-up displays, and transparent flexible displays, should block harmful UV light as well as water vapor and oxygen from reaching their materials [2,7,67,68]. Recently, a UV- and heat-reflective gas diffusion multi-barrier (UHGDM) that combines various functional layers with the respective functions has been proposed. The UHGDM was fabricated with a series of distributed Bragg reflectors (DBRs) as a UV filter, a Ag film as an IR reflector, and a GDM (Figure 5a,b). The GDM was composed of a flexible and impermeable nanolaminate film deposited by ALD and a corrosion-resistant silane-based organic/inorganic polymer (silamer) layer. According to the simulation results, the UHGDM achieved the desired optical properties by effectively blocking UV and IR wavelengths (Figure 5c). The synergistic effect between the nanolaminate and silamer layers enables the UHGDM structure to achieve the WVTR of 10^−5^ g·m^−2^·day^−1^ and to be corrosion-resistant by protecting underlying UV and IR layers in harsh environments. In the UHGDM structure, the UV filter, called DBR, which is a multi-layer structure exhibiting a large refractive index difference (*n*) between layers, was fabricated as a 3.5-dyad multi-stacked structure of 33 nm thick zinc sulfide (ZnS, *n* ≈ 2.67) and 52 nm thick lithium fluoride (LiF, *n* ≈ 1.32). The Ag film inserted between the UV filter and the GDM reflected the near-IR wavelength as a heat mirror. In a device reliability test, OSCs encapsulated with the UHGDM showed excellent reliability without UV-induced degradation, although they showed a slight decrease in electrical performance due to the decrease in transmittance caused by the UHGDM (Figure 5d). As shown in the series of studies on functional encapsulation technologies from heat-transferable TFE to UHGDM, functional encapsulations should be continuously developed to commercialize low-cost flexible OEDs and to solve reliability issues related to environmental factors, such as reactive gases, UV light, and heat.

## 4. Corrosion-Resistant Wearable Encapsulations Using Chemical Bonding at the Interface between Thin Films

**Figure 6:**
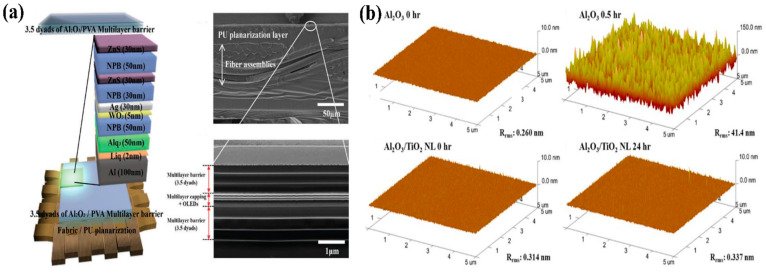
(**a**) Schematic structure and cross-sectional SEM images of the proposed fabric-based OLEDs with planarization layers and multi-layer barrier films. Reproduced with the permission of Reference [5]. Copyright 2016, Wiley-VCH GmbH. (**b**) AFM image and change in surface roughness of Al_2_O_3_ and Al_2_O_3_/TiO_2_ nanolaminate films after immersion in water at 90 °C. Reproduced with the permission of Reference [33]. Copyright 2014, American Chemical Society.

As the demands for wearable OLEDs formed on fabric and fibers increase beyond those for flexible OLEDs, the improvement of wearable encapsulation developments has been required to secure the reliability of wearable OLEDs. The wearable encapsulation film must have encapsulation performance to perfectly protect the OLED from the external environment when the wearable OLED is mechanically washed in water or chemically dry cleaned. TFE for wearable OLEDs was first proposed by Kim et al. [5]. They thermally evaporated an NPB/ZnS multi-layer that was deposited for the passivation of wearable OLEDs before TFEs. The passivated NPB/ZnS multi-layer was used to protect the permeability of the H_2_O source used as a reactant for ALD Al_2_O_3_ formation (Figure 6a). Next, a 3.5-dyad Al_2_O_3_/organic multi-layer was formed for barrier performance. In addition, Kwon et al. reported fiber-based wearable OLEDs and used the single-layer Al_2_O_3_ as a TFE layer using ALD with good step coverage [69]. However, the TFEs used in the existing wearable OLEDs are composed of films that are vulnerable to moisture, resulting in degradation of the encapsulation performance over time. In particular, many studies have been conducted on the poor environmental stability of Al_2_O_3_ ALD films in harsh conditions with a high level of temperature/relative humidity [30,34,37]. The Al_2_O_3_ film was easily degraded through an active chemical reaction with H_2_O at a high temperature, resulting in a phase transition, which caused the re-crystallization of the film. The results show that corroded Al_2_O_3_ films lose their barrier and mechanical properties due to re-crystallization. As previously reported wearable encapsulations have been fabricated based on Al_2_O_3_ ALD films, it is easy to apply these to wearable encapsulations to ensure their reliability when exposed to outdoor climates and mechanical stress. Therefore, a corrosion-resistant inorganic layer should be developed, or a corrosion-protective organic layer should be introduced to protect the inorganic layer.

First, over the years, attempts have been made to fabricate corrosion-resistant inorganic GDB layers. The corrosion-resistant properties of GDBs are also directly related to the lifetime of TFE layers. For commercialization of the display panels, they must pass the harsh reliability test of 85 °C/85% relative humidity without pixel shrinkage or dark spots. Therefore, it is necessary to improve the stability and lifetime of TFE layers for practical applications in OLEDs. As previously discussed (details can be found in Section 2), although the ALD nanolaminate system has been proposed to fabricate highly impermeable and flexible GDBs, the nanolaminate structure can also improve environmental stability by forming aluminate phases at the interfaces between Al_2_O_3_ and other oxide sublayers [34,37,47]. Meyer et al. were the first to discover the origin of the corrosion-resistant properties of the Al_2_O_3_/ZrO_2_ nanolaminate structure using X-ray photoemission spectroscopy (XPS) analysis. The 100 nm thick Al_2_O_3_ single layer and Al_2_O_3_ (95 nm)/ZrO_2_ (5 nm) bilayer showed clear barrier properties and environmental reliability in Ca tests. The addition of ZrO2 effectively protected the underlying Al_2_O_3_ layer from harsh environments. The XPS spectra of Al_2_O_3_, ZrO_2_, and Al_2_O_3_/ZrO_2_ showed a shift in the energy level of Al and Zr cores. The binding energy shift is most likely due to differences in the electronegativities of Al (1.61) and Zr (1.33). After the demonstration and significant analysis of the Al_2_O_3_/ZrO_2_ nanolaminate, Meyer et al. developed the Al_2_O_3_/HfO_2_, AT, and ZAM through material design. Kim et al. experimentally compared the environmental stability of the AT nanolaminate structure with that of the Al_2_O_3_ film. The proposed AT nanolaminate showed almost unchanged surface roughness and surface SEM images after immersion in hot water (Figure 6b). The Al_2_O_3_ and TiO_2_ sublayers formed the aluminate phase at the interface by alternately forming Al (1.61) and Ti (1.54) based on the electronegativity differences. In other words, AT nanolaminate structure shows the possibility of application as a barrier film for wearable encapsulation (Figure 6b). As a similar study, Kwon et al. also developed a highly impermeable, robust, and flexible TFE method by optimizing the thickness of the ZAM nanolaminate and confirmed the changes in WVTR in harsh environments [41]. Therefore, the functional design of GDBs makes nanolaminate impermeable, flexible, and environmentally stable.

**Figure 7:**
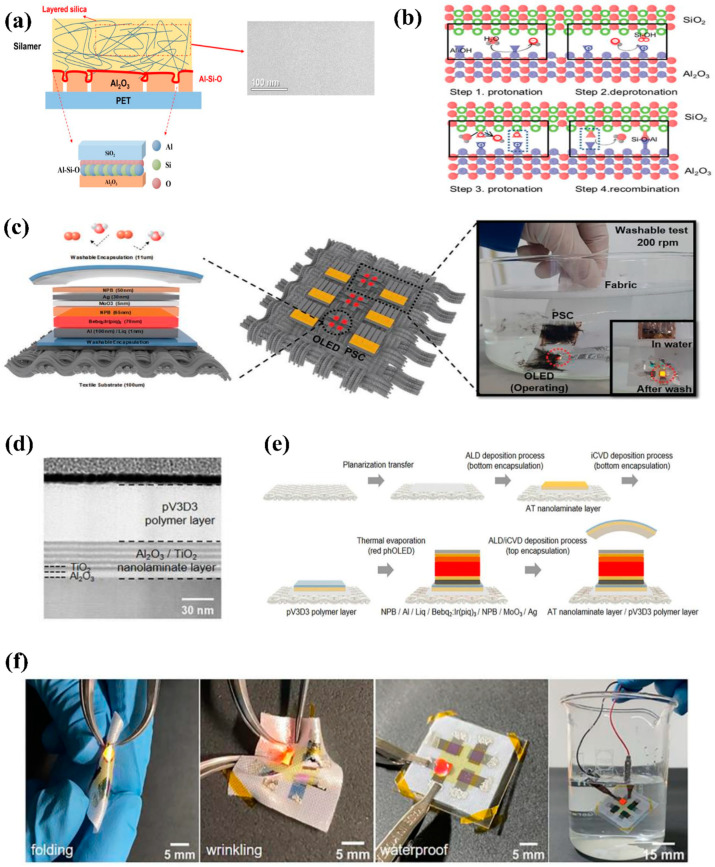
Corrosion-resistant encapsulation structure. (**a**) Chemical bonding at the interface between Al_2_O_3_ and silamer. Reproduced with the permission of Reference [29]. Copyright 2019, American Chemical Society. (**b**) Formation mechanism of the aluminate phase between the organic layer and Al_2_O_3_. (**c**) Schematics of the optoelectronic module with OSCs and OLEDs and a photograph of the operating module during and after washing. Reproduced with the permission of Reference [12]. Copyright 2019, Royal Society of Chemistry. (**d**) TEM image of the encapsulation layer. (**e**) Fabrication process of textile-based OLEDs. (**f**) From left to right, photograph of the hand folded textile-based OLED, hand wrinkled textile-based OLED, and after and during immersion in water. Reproduced with the permission of Reference [32]. Copyright 2021, Nature Publishing Group.

Second, attempts have been made to prevent degradation of the inorganic layer by using functional organic layers in a multi-barrier system [12,29]. Organic layers in TFEs have been used for surface planarization, stress relaxation, and the defect-decoupling effect between inorganic GDBs. Recently, Kwon et al. have reported highly impermeable and corrosion-resistant TFEs based on the inorganic–organic hybrid polymer with randomly formed three-dimensional silica layers for wearable encapsulations. The polymer layer forms the aluminate phase at the interface with the Al_2_O_3_ films, and the chemical reaction at the interface enables the TFE to be corrosion-resistant against water (Figure 7a). Furthermore, Jeong et al. explained the corrosion mechanism of ALD Al_2_O_3_ films and the formation mechanism of the aluminate phase at the interface between the SiO_2_-based polymer and Al_2_O_3_ in detail. Figure 7b illustrates the chemical mechanism by which the aluminate phase formed between the SiO_2_-based polymer and Al_2_O_3_ inhibits phase transition, as explained theoretically. At the interface of the polymer having high SiO_2_ content and the ALD Al_2_O_3_ surface, one or more silicification reactions lead to Al–O–Si chemical bonding and thus prevent phase transition. During water and damp heat immersion tests for reliability comparison of several kinds of organic layers, the developed wearable encapsulation exhibited meaningful improvement in environmental stability, and hardly any changes in WVTRs and surface roughness induced by GDB degradation were observed. Based on these experimental results, the reported wearable TFEs demonstrated the potential for practical realization of optoelectronic devices on real textiles without performance degradation after washing (Figure 7c). Recently, interesting research on highly flexible and corrosion-resistant wearable TFE using ALD nanolaminate and initiated-CVD-based polymer film has been conducted [32]. As shown in Figure 7d, the bilayer structure, which showed the mechanical robustness to withstand 1.7% strain and environmental stability, with its WVTR changing within one order of magnitude even after immersion in water for seven days, was fabricated through the deposition of the highly cross-linked polymer film on the AT nanolaminate film. The entire fabrication process of the encapsulated OLEDs on textile is similar to that of conventional flexible OLEDs, and the wearable OLED proved to perform well even when subjected to various types of mechanical stress or directly exposed to water owing to the excellent performance of the wearable TFE (Figure 7e,f). Wearable OLEDs will become a reality when a wearable encapsulation film that can guarantee high reliability in extreme environments, such as chemical dry cleaning and wet cleaning where mechanical stress is applied, along with surface planarization technology on a wearable substrate such as textile or fiber, is developed.

## 5. Future Works for the Development of Thin Film Encapsulation Technology

Since Vitex’s Barix encapsulation—TFE, wherein an organic layer and an inorganic layer are alternately stacked—was first reported, encapsulation techniques reported to date have aimed to improve the reliability of wearable and flexible OLEDs. For improving the mechanical robustness of encapsulation structures, stress engineering approaches, such as developing inorganic membranes using nanolaminate and crack arrestor techniques, controlling the neutral axis by inserting an additional buffer layer, and maintaining zero residual stress in the structure, have been employed. In addition, a new trend in encapsulation research is functional TFEs; these films are fabricated to provide tailored solutions to the technical issues of different types of displays. Although these TFEs have potential, their functional performance in terms of heat dissipation, out-coupling effect, corrosion resistance, harmful light blocking capacity, etc., needs to be improved. Future studies on TFEs should aim to improve their performance through structural and material improvements.

**Figure 8:**
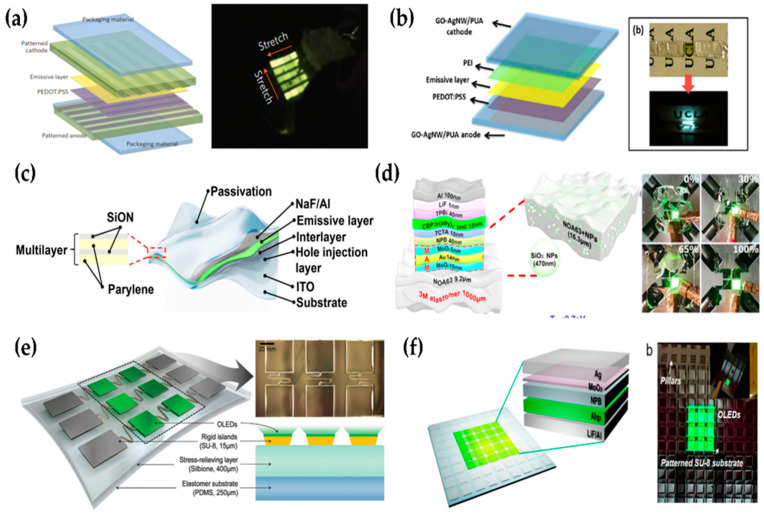
Various types of stretchable OLEDs. (**a**) Schematic illustrations of an encapsulated fully stretchable elastomeric polymer light-emitting device (EPLED) display (**left**) and demonstrations of EPLED displays being stretched (**right**). Reproduced with the permission of Reference [70]. Copyright 2013, Nature Publishing Group. (**b**) Schematic drawing of a fully stretchable PLED using GO-AgNW/PUA composite electrode as both anode and cathode. Reproduced with the permission of Reference [75]. Copyright 2014, American Chemical Society. (**c**) Structure of the ultraflexible PLED. The passivation layer was composed of alternating organic (500 nm thick parylene) and inorganic (200 nm thick SiON) layers. Reproduced with the permission of Reference [72]. Copyright 2016, American Association for the Advancement of Science. (**d**) The schematic illustration of the geometrically stretchable organic light-emitting diodes device structure along with thickness of the layers. Reproduced with the permission of Reference [79]. Copyright 2021, Nature Publishing Group. (**e**) Schematic diagrams of the proposed hybrid stretchable platform, on which OLEDs are deposited. Both tilted top-view and cross-sectional view diagrams are provided. The inset photograph on the top right side shows the microscope image of SU-8-based rigid islands and serpentine interconnectors fabricated on top of a Silbion layer. Reproduced with the permission of Reference [78]. Copyright 2020, Wiley-VCH GmbH. (**f**) Compositional schematic of the top-emitting green OLED device on a stretchable substrate (**left**) and optical image of stretchable OLEDs, indicating each component. Reproduced with the permission of Reference [76]. Copyright 2020, American Chemical Society.

In addition to flexible and wearable encapsulations, another research avenue is stretchable encapsulation technology for the realization of stretchable displays, which are considered as the ultimate displays. The stretchable TFE technology is considered to be the most difficult to the extent that proper research results have never been published. Various types of stretchable OLEDs, such as intrinsically stretchable OLEDs (ISOLEDs), pre-strained elastomer-attached OLEDs (PEOLEDs), and geometrically stretchable OLEDs (GSOLEDs), have been previously demonstrated [70,71,72,73,74,75,76,77,78,79,80]. However, the encapsulation structures for these stretchable OLEDs were one-layer elastomer or conventional organic–inorganic TFE structures. ISOLEDs are fabricated with or without one layer of elastomer applied as a TFE structure (Figure 8a,b). In general, organic layers comprising polymers and elastomers have poor WVTR of more than 1 g·m^−2^·day^−1^ despite their high thickness [81,82]. In other words, such a layer can hardly act as an encapsulation layer that protects the OLED. As metal oxide layers cannot be intrinsically stretched owing to their physical structure, they cannot be applied to ISOLEDs. Kim et al. reported the mechanical properties of a 100 nm thick ALD Al_2_O_3_ film, which is widely used as an inorganic layer for TFE structures [83]. The ALD Al_2_O_3_ films were fractured at tensile strains (<0.5%) regardless of the deposition temperature. PEOLEDs are technically easier to fabricate than ISOLEDs because compressive stress acts as a major stress applied to devices (Figure 8c,d). This is because, in general, materials have better mechanical resistance to compressive stress than to tensile stress of the same value. The change in the physical properties of the thin film according to the stress type can be explained by the stress concentration theory [84]. In other words, encapsulation can ensure better reliability. Yokota et al. reported a PEOLED encapsulated with an inorganic–organic multi-barrier [72]. However, although they showed the stable operation of the PEOLED after a cyclic stretching test, data on WVTR change owing to strain were not presented. Based on the two types of OLEDs introduced above, the realization of stretchable OLEDs may be difficult in terms of long-term reliability because a TFE layer composed of inorganic layers is mechanically damaged by direct pulling or compression, resulting in an increase in WVTR. To realize the ultimate ISOLED, an encapsulation layer composed of an organic–inorganic hybrid elastomer and having self-healing effect should be developed. The hybrid elastomer should exhibit excellent elasticity and a significant decrease in WVTR as the thickness increases. GSOLEDs are fabricated on elastomer islands free from mechanical stress because the serpentine interconnectors connecting the islands are stretched while the islands are fixed, as shown in Figure 8e,f [76,78]. Although GSOLEDs have limitations such as the high-resolution patterning issue, degree of stretching, and conspicuous non-emitting area, they are the easiest and most effective method to realize stretchable OLEDs. However, for highly reliable GSOLEDs, the OLED and TFE layer should be formed only on the elastomer islands. When layers formed on the interconnectors are fractured and delaminated by repetitive stretching, these fractures can propagate to the layers formed on elastomer islands. Therefore, techniques such as area-selective ALD and laser patterning are suggested for OLED fabrication on elastomer islands.

## 6. Conclusions

This review highlights the technical issues that require consideration for the realization of reliable flexible OLEDs and discusses various technical attempts to solve these issues through material and structural design. As OLEDs are considered the most promising candidates for the realization of next-generation displays beyond liquid-crystal displays, research on encapsulation technologies has been actively conducted. The initial TFE technology focused on achieving excellent barrier performance by increasing the number of organic/inorganic layer pairs or by increasing the layer thickness. However, as flexible displays move from curved displays to foldable/rollable displays, the required target specifications of the TFE have increased, necessitating the development of an inorganic layer that greatly affects the barrier and mechanical properties of the TFE. With the advent of ALD, low-temperature, high-quality thin-film deposition, and angstrom-level structural design became possible, and as a result, the main performance of TFEs was significantly enhanced. Furthermore, through the application of stress engineering, such as NA control, zero internal stress, and a nanolaminate structure, the mechanical reliability of the encapsulation film and OLED has been improved to a level where foldable/rollable displays can be realized. In recent years, as the development of wearables and transparent displays has progressed, various multi-functional encapsulation technologies have been proposed to solve reliability issues related to environmental factors such as moisture/oxygen, solar radiation, and corrosion. From a transparent functional encapsulation film that blocks UV and IR rays to a washable wearable encapsulation, the development of an encapsulant customized to the characteristics of a display will enable the commercialization of displays. Considering the development history of OLEDs, the most vital technology for the commercialization of OLEDs was the encapsulation technology that guarantees the lifetime and reliability of the devices. In the future, for the realization of stretchable OLEDs, which is considered to be the most technically challenging beyond current display technology, it is necessary to focus on the development of stretchable encapsulation technologies based on material and structural design and ideas that go beyond the existing framework.

## Figures and Tables

**Table 1:** WVTR and mechanical and environmental reliability test of TFEs based on various ALD nanolaminate films.

ALD Nanolaminate	Achieved WVTR [g·m^−2^·day^−1^] (Test Condition)	Bending Test [g·m^−2^·day^−1^] (Bending Radius or Strain)	Environmental Stability Test (to Test or Not to Test)	Device Characteristics after Bending (Bending Radius or Strain)	Ref.
Al_2_O_3_/SiO_2_	4.2 × 10^−5^ (25 °C/40%)	X	X	X	[40]
Al_2_O_3_/ZrO_2_	4.7 × 10^−5^ (70 °C/70%)	X	X	X	[36]
Al_2_O_3_/SiO_2_	3.8 × 10^−5^ (20 °C/50%)	1.64 × 10^−3^ (1 cm)	X	X	[38]
Al_2_O_3_/TiO_2_	1.81 × 10^−4^ (60 °C/90%)	X	O	X	[33]
Al_2_O_3_/alucone	7.10 × 10^−5^	9.94 × 10^−5^ (0.105 mm)	X	X	[31]
Al_2_O_3_/MgO	4.6 × 10^−6^ (60 °C/100%)	X	O	X	[43]
Al_2_O_3_/SAOLs	1.58 × 10^−3^ (85 °C/85%)	3.81 × 10^−3^ (1 cm)	X	X	[45]
Al_2_O_3_/ZnO	7.87 × 10^−6^ (30 °C/90%)	7.78 × 10^−5^ (1 cm)	X	1 cm	[44]
Al_2_O_3_/ZnO/MgO	2.44 × 10^−6^ (30 °C/90%)	9.78 × 10^−5^ (0.6 cm)	O	0.8 cm	[41]
Al_2_O_3_/MgO	1.70 × 10^−^^5^ (30 °C/90%)	9.78 × 10^−5^ (1.25%)	O	0.63%	[47]
Al_2_O_3_/TiO_2_	9.94 × 10^−6^ (30 °C/90%)	~10^−5^ (1.7%)	O	0.15 cm	[32]

## Data Availability

Not applicable.
